# Microeukaryotic plankton evolutionary constraints in a subtropical river explained by environment and bacteria along differing taxonomic resolutions

**DOI:** 10.1093/ismeco/ycae026

**Published:** 2024-02-24

**Authors:** Kexin Ren, Yuanyuan Mo, Peng Xiao, Regin Rønn, Zijie Xu, Yuanyuan Xue, Huihuang Chen, Windell L Rivera, Christopher Rensing, Jun Yang

**Affiliations:** Aquatic EcoHealth Group, Key Laboratory of Urban Environment and Health, Fujian Key Laboratory of Watershed Ecology, Institute of Urban Environment, Chinese Academy of Sciences, Xiamen 361021, China; Aquatic EcoHealth Group, Key Laboratory of Urban Environment and Health, Fujian Key Laboratory of Watershed Ecology, Institute of Urban Environment, Chinese Academy of Sciences, Xiamen 361021, China; Key Laboratory of Urban Environment and Health, Ningbo Urban Environment Observation and Research Station, Institute of Urban Environment, Chinese Academy of Sciences, Xiamen 361021, China; Zhejiang Key Laboratory of Urban Environmental Processes and Pollution Control, CAS Haixi Industrial Technology Innovation Center in Beilun, Ningbo 315830, China; Aquatic EcoHealth Group, Key Laboratory of Urban Environment and Health, Fujian Key Laboratory of Watershed Ecology, Institute of Urban Environment, Chinese Academy of Sciences, Xiamen 361021, China; National and Local Joint Engineering Research Center for Ecological Treatment Technology of Urban Water Pollution, College of Life and Environmental Science, Wenzhou University, Wenzhou 325035, China; Aquatic EcoHealth Group, Key Laboratory of Urban Environment and Health, Fujian Key Laboratory of Watershed Ecology, Institute of Urban Environment, Chinese Academy of Sciences, Xiamen 361021, China; Department of Biology, University of Copenhagen, Copenhagen DK2100, Denmark; Aquatic EcoHealth Group, Key Laboratory of Urban Environment and Health, Fujian Key Laboratory of Watershed Ecology, Institute of Urban Environment, Chinese Academy of Sciences, Xiamen 361021, China; University of Chinese Academy of Sciences, Beijing 100049, China; Aquatic EcoHealth Group, Key Laboratory of Urban Environment and Health, Fujian Key Laboratory of Watershed Ecology, Institute of Urban Environment, Chinese Academy of Sciences, Xiamen 361021, China; Aquatic EcoHealth Group, Key Laboratory of Urban Environment and Health, Fujian Key Laboratory of Watershed Ecology, Institute of Urban Environment, Chinese Academy of Sciences, Xiamen 361021, China; University of Chinese Academy of Sciences, Beijing 100049, China; Pathogen-Host-Environment Interactions Research Laboratory, Institute of Biology, College of Science, University of the Philippines Diliman, Quezon City 1101, Philippines; Aquatic EcoHealth Group, Key Laboratory of Urban Environment and Health, Fujian Key Laboratory of Watershed Ecology, Institute of Urban Environment, Chinese Academy of Sciences, Xiamen 361021, China; Institute of Environmental Microbiology, College of Resources and the Environment, Fujian Agriculture & Forestry University, Fuzhou 350002, China; Aquatic EcoHealth Group, Key Laboratory of Urban Environment and Health, Fujian Key Laboratory of Watershed Ecology, Institute of Urban Environment, Chinese Academy of Sciences, Xiamen 361021, China

**Keywords:** plankton, river, taxonomic resolution, community ecology, bacterial diversity, environmental filtering

## Abstract

Microeukaryotic plankton communities are keystone components for keeping aquatic primary productivity. Currently, variations in microeukaryotic plankton diversity have often been explained by local ecological factors but not by evolutionary constraints. We used amplicon sequencing of 100 water samples across five years to investigate the ecological preferences of the microeukaryotic plankton community in a subtropical riverine ecosystem. We found that microeukaryotic plankton diversity was less associated with bacterial abundance (16S rRNA gene copy number) than bacterial diversity. Further, environmental effects exhibited a larger influence on microeukaryotic plankton community composition than bacterial community composition, especially at fine taxonomic levels. The evolutionary constraints of microeukaryotic plankton community increased with decreasing taxonomic resolution (from 97% to 91% similarity levels), but not significant change from 85% to 70% similarity levels. However, compared with the bacterial community, the evolutionary constraints were shown to be more affected by environmental variables. This study illustrated possible controlling environmental and bacterial drivers of microeukaryotic diversity and community assembly in a subtropical river, thereby indirectly reflecting on the quality status of the water environment by providing new clues on the microeukaryotic community assembly.

## Graphical Abstract

**Graphical Abstract:**
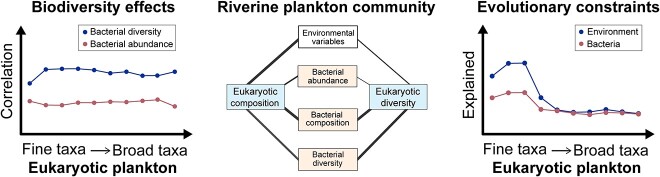

## Introduction

Eukaryotic microbes are crucial in the biosphere, as they can affect microbial populations by horizontal gene transfer or reprogramming of the host metabolism [1]. From a traditional view, evolution has been occurring over long periods of time and has not influenced short-term ecological processes [2, 3]. Yet, a recent study demonstrated that evolution may influence host fitness indirectly through ecological interactions [4]. Niches indicate the adaptation to local environment, at the same time, it can reflect the trend of environmental stressors on relevant lineages [5]. Traditionally, microbial communities are often characterized at the species level, as attribution to species has been regarded as the basic unit of biotic classification. Microbial taxa from the same lineage often display similar reactions to environmental changes [6]. A previous study had quantified the strength of community-environment correlations, but had only focused on soil and seawater prokaryotic communities and not focused on the role of microeukaryotic diversity-environment correlations according to the sequence similarity into successive levels or taxa from species to phylum levels [6]. Therefore, it is necessary to study the correlations of microeukaryotic diversity-environment relationships at the sequence similarity into successive levels or taxa from fine to broad levels.

Over the past decade, increasing research found that biodiversity had significant influence on ecosystem functions through interactions between bacteria and eukaryotes [7, 8]. There are significant gaps regarding the coexistence patterns of microeukaryotic and bacterial communities on diversity and abundance along taxonomic resolution from fine to broad levels. Bacteria have been shown to be present in diverse abundances in various aquatic ecosystems [9]. Specifically, the absolute abundance of the 16S rRNA gene ranged from 6.07×10^8^ to 1.01×10^10^ copies/L in the 24 inland water bodies of Southeast China using qPCR method [10]. In addition, a significant positive correlation was observed between abundance of microeukaryotic 18S rRNA gene and bacterial 16S rRNA gene in a subtropical reservoir [9]. This phenomenon is anticipated to significantly contribute to the evolution of eukaryotes [9], as well as to the stability and functional robustness of microbial community [1]. In addition, long-term temporal variation patterns of microbial interactions remain relatively unknown, with only a few instances of evaluating bacterial-plankton succession in oceans over time series of decades [6, 11-13]. Previous research had explored the biodiversity link to the interaction between eukaryotes and bacteria [11, 14]. However, the potential associations of the microbial community in inland aquatic ecosystems are still lacking to be explored over space and time, especially for subtropical river watershed [15, 16].

Understanding the mechanisms (e.g. abiotic and biotic factors) driving shifts in community composition is crucial in assessing microeukaryotic diversity and ecosystem stability [16-18]. This points to a crucial challenge in improving our cognition about how ecological interactions mediate biodiversity and emphasize some groups of interacting plankton from networks with coevolution forces [19]. The interplay between eukaryotic microorganisms in the control of aquatic prokaryotes is still poorly understood [20-22].

Species identification is an indispensable part of ecological studies and represents the most basic form of biological data [23]. Operational taxonomic units (OTUs) are most commonly used as a proxy for species or taxa in DNA sequence-based data. Unfortunately, species-level community data are not always available, as there are constraints on time and the expertise required to properly identify all individuals at the species level [24-26]. Moreover, different super-groups may have different ecological values because their profiles were defined within different ecoregions with limited range of environmental variables [27]. Here, the strength of microeukaryotic community-environment/bacteria coupling along taxonomic levels and within different eukaryotic super-groups is assessed, inferring importance of evolutionary forces behind community assembly.

Therefore, we hypothesize that (1) the impact of bacteria on microeukaryotic plankton communities is mediated mainly through effects of bacterial diversity and to a lesser extent by effects of bacterial abundance; (2) the microeukaryotic community has been more influenced by environmental factors than by interactions with the bacterial community; (3) the strength of community-environment/bacterial community coupling decreases with increasing taxonomic resolution because finer taxonomic groups may exhibit distinct physiological responses. To test these hypotheses, we explored the strength of microeukaryotic plankton community-environment coupling and the interaction between bacteria and microeukaryote using 100 samples collected over a five-year period in a subtropical river.

## Materials and methods

### Study area and sampling

All samples were obtained at 10 sites from both January (dry season) and July (wet season) along the Houxi River over five years (2012–2016). The Houxi River Watershed is an important water-source conservation region and located in Xiamen, Fujian province, southeast China [16, 18]. Heat-map plots showed spatiotemporal changes of environmental parameters in the Houxi River during the five-year period (Fig. S1). Specifically, water temperature (WT) ranged from 11.63 to 32.51°C. The pH ranged from 7.04 to 9.97. The dissolved oxygen (DO) ranged from 3.03 to 17.14 mg/L. The chlorophyll *a* (Chl *a*) ranged from 0.54 to 135.08 μg/L. The turbidity ranged from 0 to 686 NTU. The electrical conductivity (EC) ranged from 15.6 to 13380.0 μS/cm. The oxidation–reduction potential (ORP) ranged from 200 to 494 mV. Total carbon (TC) ranged from 0.88 to 44.87 mg/L. Total organic carbon (TOC) ranged from 0.38 to 15.78 mg/L. Total nitrogen (TN) ranged from 0.60 to 11.59 mg/L. Ammonium nitrogen (NH_4_-N) ranged from 0.001 to 11.983 mg/L. Nitrate nitrogen (NO_3_-N) ranged from 0.293 to 2.419 mg/L. Nitrite nitrogen (NO_2_-N) ranged from 0.001 to 0.744 mg/L. Total phosphorus (TP) ranged from 0.001 to 2.050 mg/L. Phosphate phosphorus (PO_4_-P) ranged from <0.001 to 1.149 mg/L. Surface water was collected following our previous study [18, 28]. That is, the surface water samples for microbial community analysis were pre-filtered using 200 μm mesh to remove large particles. Afterwards, the water was filtered by a 0.22 μm pore-size polycarbonate filters (47 mm diameter, Millipore, Billerica, MA, USA). We collected data of 15 environmental variables [29]. More details are described in the Supplementary Information.

### DNA extraction and sequencing

Membranes with plankton cells were individually sliced into small fragments. Total DNA was extracted through the FastDNA SPIN Kit (MP Biomedicals, Santa Ana, CA, USA) according to the provided instructions. To assess microeukaryotic plankton communities, a dual-index amplicon library technique was used. This method targeted the 18S rRNA gene V9 hypervariable region with primers [30] and sequences were obtained on Illumina Hiseq platform (Illumina, San Diego, CA, USA) using 2 × 150 bp paired-end approach.

### Bioinformatics for microeukaryotic plankton

Quality filtering was performed by eliminating the low-quality reads. Default settings for quality control processing were used using QIIME V1.9.1 [31]. We deleted the chimera with the UCHIME de novo algorithm [32]. After chimera checking using USEARCH, UCLUST was used to filter the representative sequences on different similarity levels in sequence from 97% to 70% (Fig. S2).

### Microeukaryotic plankton community

There are two concurrent hierarchical taxonomic systems: (i) one relies on molecular criteria to assign ranks to operational taxonomic units (OTUs) by truncating sequence similarity (e.g. at 97%, 94%, 91% levels) and (ii) the other is based on database annotation and adheres to traditional taxonomic classification (e.g. species, genus, family, etc.) [6, 33]. Here, we employed both hierarchical systems to generate compositional profiles spanning a range of taxonomic resolutions, and examined how the intensity of community-environment relationships changed across different taxonomic ranks (Figs S2 and S3). Specifically, microeukaryotic OTUs were blasted against PR2 database (Release 115) [34, 35]. Following the exclusion of singleton, doubleton, mitochondrial, chloroplast sequences, and unknown organisms, we remained 30 897, 30 896, 30 847, 30 886, 30 879, 30 884, 30 889, 30 893, 30 876, 30 891 sequences for each sample with normalization from 97%, 94%, 91%, 88%, 85%, 82%, 79%, 76%, 73% and 70%, respectively (Fig. S2). In terms of super-group composition, communities were divided into nine categories (Alveolata, Amoebozoa, Apusozoa, Archaeplastida, Excavata, Cryptista, Opisthokonta, Rhizaria, and Stramenopiles) and unassigned taxa [8, 36]. Note that the super-group Apusozoa was not included in the follow-up analyses due to its very minor contribution to the microeukaryotic plankton communities (Fig. S3).

### Bacterial community

The DNA from the total 100 samples were amplified separately using universal bacterial primers (341F and 806R) that target the V3–V4 hypervariable regions of the 16S rRNA gene [16]. The OTU table of bacterial community was got based on 97% similarity level [34]. Other bioinformatics were processes similar to 18S rRNA gene [16].

Using an Illumina HiSeq platform, gene fragments underwent sequencing employing a paired-end approach. Following quality assessment, amplicon sequences were grouped into operational taxonomic units (OTUs) using the “unoise3” component of the USEARCH tool [32]. The resulting OTU table was generated with a 97% similarity threshold, using the VSEARCH software [34].

### Real-time quantitative PCR

The abundances of 16S rRNA and 18S rRNA genes were evaluated using real-time quantitative PCR on a Light Cycler 480 (Roche, Basel, Switzerland). The process of PCR amplification was conducted in triplicate, employing a thermal profile involving an initial phase of 30 seconds at 94°C, succeeded by 40 cycles encompassing 5 seconds at 94°C, 15 seconds at 50°C, and 10 seconds at 72°C. The reaction composition comprised 20 ng of template DNA, 0.6 μM for each primer, and 2 × SYBR premix Ex Taq II. To determine the absolute abundance of the 16S rRNA and 18S rRNA genes, we applied a six-point calibration curve from a 10-fold dilution of a standard plasmid containing a cloned and sequenced 16S rRNA or 18S rRNA gene fragment starting with the highest concentration, respectively. The amplification efficiency (*E*) was estimated using the slope of the standard curve through the following formula: *E* = (10^–1/slope^)^−1^. After evaluation of the analysis parameters, the standard curves were r^2^ = 0.99 and the efficiency of PCR was between 95% and 105% for 16S rRNA and 18S rRNA genes in this study.

### Statistical analyses

To quantify the strength of the correlation between microeukaryotic and bacterial communities/environmental factors along taxonomic rank, the distance-based redundancy analysis (dbRDA) [37] was used except for the super-group Apusozoa and Amoebozoa, because the richness of these groups is too low to meet the calculation requirements. Further, to explore the synchronicity of plankton communities, Procrustes analysis was executed to ascertain the interrelation between each set of multi-dimensional scaling ordination plots.

We transformed the environmental variables into a set of orthogonal values using principal coordinate analysis (PCoA). The PCoA first axis, explaining 88.6% of the total variance, was employed as a surrogate for depicting the comprehensive environmental heterogeneity. To assess bacterial community dissimilarity, nonmetric multidimensional scaling (NMDS) of the Bray–Curtis distance matrix pertaining to OTU-level community composition was conducted. The first axis of NMDS was used for further analyses. For microeukaryotic plankton community, we calculated community dissimilarity using NMDS, then the first axis (NMDS first axis: NMDS1) was used from 97% to 70% similarity levels, respectively. NMDS1 refers to the first axis or dimension resulting from the NMDS analysis. That is, NMDS1 represents the relative positions of samples along the first axis of the lower-dimensional space, which represent compositional gradients. This axis is derived by reducing the original high-dimensional data while preserving the pairwise dissimilarities or similarities between samples [38, 39].

### Co-occurrence network

An integrated network was established using a dataset encompassing 100 samples spanning the entire study duration. Additionally, eight distinct sub-networks were constructed between bacteria and microeukaryotes, each aligned with a super-group of microeukaryotes based on annotations. We restricted our analysis to taxa presence in ≥1/3 of the samples to reduce noise and thus false-positive predictions. Spearman correlation was employed to explore associations shared between microeukaryotic and bacterial taxa. Only correlations meeting stringent criteria—being both robust (|r| ≥ 0.6) and statistically significant (*P* < 0.01) were integrated into the network analyses [40, 41].

Spearman correlation was applied to examine the correlations between bacterial richness (number of OTUs)/bacterial community composition (NMDS first axis)/bacterial abundance (16S rRNA gene copy number)/environmental variables (PCoA first axis) and microeukaryotic richness (number of OTUs)/microeukaryotic community composition that were calculated from 97% to 70% similarity levels. Furthermore, we only kept the significant correlations among the environmental variables (*P* < 0.05). Spearman’s correlations were calculated using the “picante” package in R [42].

## Results

### The effects of bacterial diversity and abundance on microeukaryotic plankton community along taxonomic resolution

**Figure 1:**
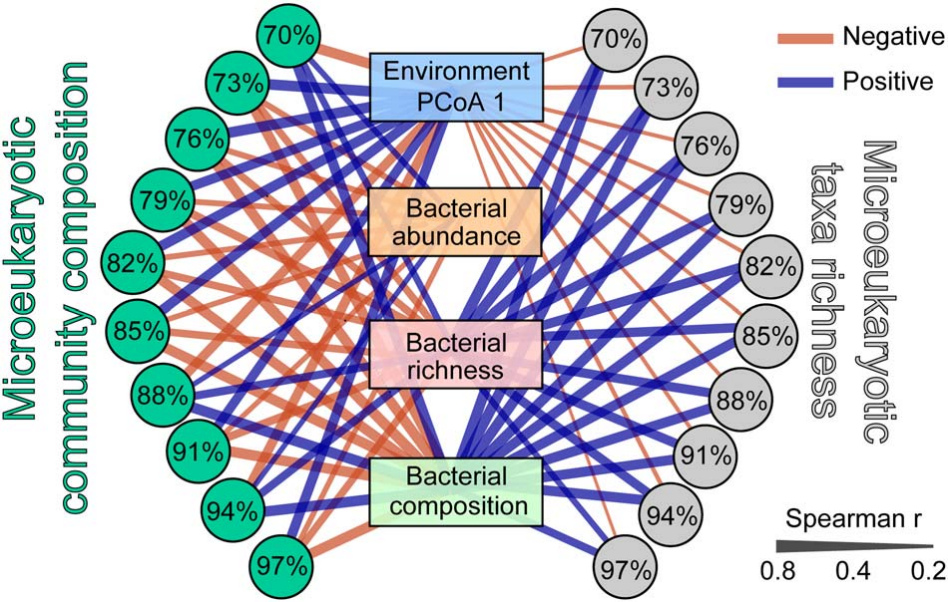
Microeukaryotic taxa richness exhibited strong correlations with bacterial richness and bacterial community composition from fine to broad taxonomic resolutions, while microeukaryotic plankton community composition displayed a strong correlation with environmental variables and bacterial community along fine to broad taxonomic resolutions. The microeukaryotic plankton community composition was estimated as the axis 1 of nonmetric multidimensional scaling (NMDS) at the OTU-level community composition and taxa richness was defined as number of different OTUs from 97% to 70% sequence-similarity levels. The environmental variable was estimated by combining 15 environmental factors into one variable by principal coordinate analysis; bacterial abundance was calculated by 16S rRNA gene copies/L; bacterial richness was defined as numbers of bacterial OTUs at 97% sequence-similarity level; bacterial composition represents the value of the NMDS1 from the bacterial community. Line width shows the correlated strength, orange line and blue line represent negative and positive correlations, respectively.

We detected significant correlations between environmental variables (PCoA 1), bacterial abundance, bacterial richness, bacterial community composition and microeukaryotic plankton community composition or species richness from fine to broad taxonomic resolutions (Fig. 1). The microeukaryotic taxa richness displayed a stronger linkage to bacterial richness and community composition than bacterial abundance. Meanwhile, apart from 70%, 88%, and 94% similarity levels, the microeukaryotic plankton community compositions showed a positive correlation to the environment (Fig. 1).

From these 100 samples, a total of 90 080, 34 334, 20 786, 14 091, 9801, 7054, 5087, 3650, 2558, and 1784 microeukaryotic OTUs were obtained at 97%, 94%, 91%, 88%, 85%, 82%, 79%, 76%, 73%, and 70% sequence similarity, respectively (Fig. S2). Overall, the most abundant group was corresponding to Opisthokonta at 97% sequence similarity level (Fig. S2). In all ten cases, the diversity of communities weighted by abundance measured at different taxonomic resolutions correlated significantly to similarities at other taxonomic levels (Fig. S3). For example, similarity at 97% and 94% levels roughly corresponded to species-genus levels, with Procrustes correlations ≥0.8 in most cases (Fig. S3); similarity at 79% and 70% levels were comparable to the phylum level. Based on Procrustes analysis, our approach displayed no differences between different similarity levels or annotation taxa in total microeukaryotic plankton community composition (Fig. S3). Furthermore, some groups of eukaryotes (Alveolata, Amoebozoa, Excavata, Cryptista, Opisthokonta, Rhizaria, and Stramenopiles) displayed a weak correlation between sequence similarity and database annotation, respectively (Fig. S3).

**Figure 2:**
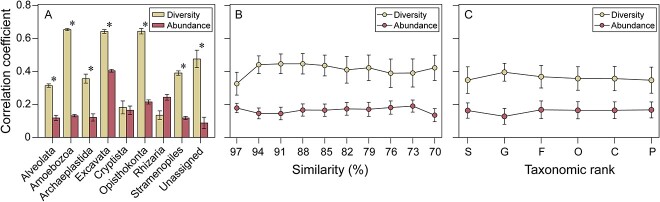
Spearman correlations between microeukaryotic plankton diversity and bacterial diversity or abundance measured at different eukaryotic supergroups, taxonomic and phylogenetic resolutions. (A) Bar-plot summarizing the strength of the correlation between microeukaryotic plankton richness (the number of OTUs), bacterial diversity (richness) and abundance (log 16S rRNA gene copies) for the different microeukaryotic supergroups. All correlations between microeukaryotic plankton diversity and bacterial diversity/abundance were significant (*P* < 0.01). Asterisks denote a significant difference between the strength of the effect of bacterial diversity and bacterial abundance on microeukaryotic plankton diversity by two-sample t-tests (^*^P < 0.05). (B) Correlation coefficients between microeukaryotic plankton diversity and bacterial OTUs and abundance calculated for microeukaryotic OTUs defined at different levels of sequence similarity. (C) Correlation coefficients between microeukaryotic plankton diversity and bacterial diversity/abundance along the taxonomic rank from species to phylum levels (S: Species, G: Genus, F: Family, O: Order, C: Class, P: Phylum) based on database annotation.

The Spearman’s correlation analysis showed significant positive correlations between microeukaryotic diversity (the number of eukaryotic OTUs) and bacterial diversity (richness) or abundance (bacterial 16S rRNA gene copies) (Fig. 2). The significant positive correlation was also found between microeukaryotic abundance (microeukaryotic 18S rRNA gene copies) and bacterial abundance (bacterial 16S rRNA gene copies) (Fig. S4). We further calculated the correlation between richness (number of OTUs) of the nine different eukaryotic groups and bacterial abundance. Both Cryptista and Rhizaria showed no significant difference between the correlation with bacterial OTUs and the correlation with bacterial gene copies (Fig. 2A), but there were significantly higher effects of bacterial species richness for the other seven groups (Alveolata, Amoebozoa, Archaeplastida, Excavata, Opisthokonta, Stramenopiles, and unassigned taxa) (Fig. 2A). The richness of the microeukaryotic plankton community from fine to broad taxonomic resolution was also more affected by bacterial richness than by bacterial abundance (Fig. 2B and 2C). Shannon-Wiener index revealed a significantly higher effect of bacterial diversity than of bacterial abundance on the diversity of the groups Amoebozoa, Archaeplastida, Excavata, Opisthokonta, Stramenopiles, and unassigned eukaryotes (Fig. S5A). The bacterial diversity exhibited a stronger correlation coefficient with microeukaryotic diversity than bacterial abundance from fine to broad taxonomic resolution (Fig. S5B) and phylogenetic resolution (Fig. S5C).

### The influence of evolutionary constraints on environmentally-explained plankton community variance was higher than for the bacterial community

**Figure 3:**
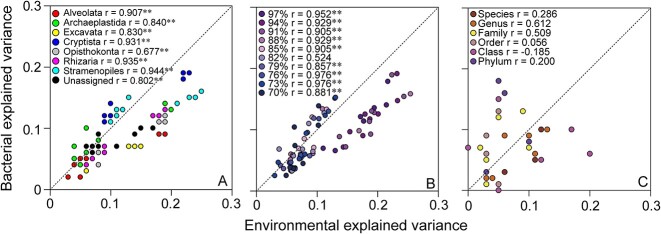
Microeukaryotic plankton community composition variation explained by environmental variables and the bacterial community, respectively. (A) Distance-based redundancy analysis (dbRDA) was employed to quantify the impact of environments and bacteria on the community compositions of distinct microeukaryotic super-groups, as indicated by the adjusted r^2^ representing the explained variance. (B) Based on sequence similarity, a range of taxonomic levels were considered, and distance-based redundancy analysis (dbRDA) was applied to measure the effect of environmental factors and bacterial community on microeukaryotic plankton composition. The adjusted R^2^ value represented the proportion of variance explained. (C) the interplay between environments or bacterial community and microeukaryotic plankton (i.e. adjusted R^2^, the explained variance) was assessed through distance-based redundancy analysis (dbRDA) from species to phylum levels based on database annotation. The letter “r” represents the correlation between environmental explained variance and bacterial explained variance. ^*^P <0.05, ^**^P < 0.01.

The dbRDA demonstrated a significant impact of environmental factors and bacterial community on the community variation in different microeukaryotic super-groups, taxonomic and phylogeny resolutions, respectively (Fig. 3). Microeukaryotic super-groups and phylum taxa were obtained based on PR2 database annotation from microeukaryotic OTU table at 97% similarity level. Across all datasets, eight super-groups displayed a significant correlation between the effects of the environmental variables and the bacterial community (Fig. 3A). More importantly, at fine taxonomic resolution (91%–97% microeukaryotic plankton community composition), there were higher explained variance by environments compared to bacterial community (Fig. 3B), suggesting that environmental selection is a strong force determining microeukaryotic plankton community at fine taxonomic level. These results indicated that environmental factors and bacterial community have a distinct influence on the microeukaryotic plankton community. In contrast, the correlation between environment and bacterial community based on microeukaryotic community composition from species to phylum levels showed no significant relationship (Fig. 3C).

From the integrated co-occurrence network, we identified 10 653 positive and 503 negative significant (*P* <0.05) co-occurrences between microeukaryotic and bacterial taxa (Fig. S6). We also found positive co-occurrences between Alveolata and bacteria (58), Amoebozoa and bacteria (3), Archaeplastida and bacteria (152), Excavata and bacteria (225), Cryptista and bacteria (35), Opisthokonta and bacteria (248), Rhizaria and bacteria (3), and Stramenopiles and bacteria (152) (Fig. S6). In addition, negative co-occurrences of Archaeplastida and bacteria (33), Excavata and bacteria (42), Cryptista and bacteria (25), Opisthokonta and bacteria (87), Rhizaria and bacteria (1), and Stramenopiles and bacteria (33) could be observed (Fig. S6). Based on network analysis, the top five interacting OTUs were depicted (Fig. S7). We noted that when the microeukaryotic OTUs peaked in relative abundance, it would be significantly regulated via the members of Firmicutes, Actinobacteria, and Proteobacteria in the bacterial community (Fig. S7).

### Niche-related signals of the microeukaryotic plankton community response to either descending or remaining stable along taxonomic ranks

**Figure 4:**
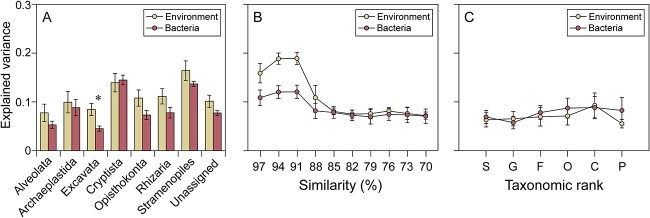
Change in microeukaryotic plankton community composition quantified via environmental variables and the bacterial community. The level of explained variance (adjusted R^2^) was explained using distance-based redundancy analysis (dbRDA). (A) Explained variance for the eight major microeukaryotic groups from microeukaryotic plankton OTU table based on 97% similarity level. (B) Explained variance for the microeukaryotic plankton communities at different levels of sequence similarity (from 97% to 70%). (C) Explained variation for the microeukaryotic plankton communities at different phylogenetic resolutions based on database annotation (S: Species, G: Genus, F: Family, O: Order, C: Class, P: Phylum). Asterisks denote the significant difference between the strength of the effect of environmental variables and bacterial community on microeukaryotic plankton community by two-sample t-tests (^*^P < 0.05). Bacteria represent the value of the NMDS1 from the bacterial community.

The dbRDA was performed for explaining the microeukaryotic plankton community compositions (Fig. 4). The explained variance tended to decrease or keep stable with broadening taxonomic resolution based on the total microeukaryotic plankton community (Fig. 4). In general, eukaryotic groups were better explained by environmental factors than the bacterial community except Cryptista (Fig. 4A). Alongside taxonomic resolution, the explained variance gradually increased from 97% to 91% similarity levels, then decreased to a lower level at 88% similarity level and finally approached invariance from 85% to 70% similarity levels (Fig. 4B). However, the explained strength of the environment and the bacterial community on microeukaryotic plankton community displayed no clear trend along different phylogenetic resolutions from species to phylum levels (Fig. 4C).

Mantel test indicated that Alveolata and Excavata were significantly correlated with pH. Archaeplastida were significantly correlated with pH, TN and NO_2_-N. Rhizaria were significantly correlated with pH, TC, NO_3_-N, and NO_2_-N. Stramenopiles were significantly correlated with Chl *a*, TC, TN, NO_2_-N, and TP. Unassigned taxa were significantly correlated with NO_3_-N (Fig. S8).

## Discussion

### Bacterial diversity had a greater impact on microeukaryotic plankton diversity than its abundance

In the river ecosystem, biodiversity increased with bacterial richness in eukaryotic groups along fine to broad taxa rather than being the effect of bacterial abundance (Fig. 2). The Archaeplastida are a major group of autotrophic eukaryotes, comprising Chlorophyta, Glaucophyta, Rhodophyta, and Streptophyta. The correlation between bacterial diversity and microeukaryotic plankton diversity varied between the different microeukaryotic plankton groups. These may reflect their distinct feeding or predation patterns [43-45]. The bacterial diversity also significantly affected the community diversity of the three groups mainly comprised of bacterivorous protists (Alveolata, Amoebozoa, and Excavata) (Fig. 2A). The coupling between the diversity of bacteria and bacterivorous protists is likely to be highly complex and the three groups will mutually affect each other. Bacteria normally vary considerably in their ability to resist and avoid predation by protists. Hence, predation poses a strong selection factor affecting survival of bacteria in the environment and distinct strategies have emerged in bacteria to avoid predation by other taxa [46, 47]. Previous microcosm studies have shown that the presence of protists significantly affected bacterial community composition [48].

Our results also partly confirmed that higher bacterial diversity was associated with the higher microeukaryotic plankton diversity, whereas bacterial abundance displayed significant but lower effects (Fig. 2), even if bacterial abundance was positively correlated to microeukaryotic plankton abundance (Fig. S4). The close coupling between the taxonomic diversity of microbial communities was displayed in coastal and model ecosystems [49, 50]. Conversely, some previous studies were performed in closed ecosystems, which excluded the possibility of immigration [13]. Our study area allowed microbial activity, which may have diminished the impact of predators on prey via compensating prey biomass [51, 52]. Among the protists that feed on bacteria, the major predators of freshwater bacteria were widely from heterotrophic nanoflagellates and ciliates [53, 54]. Meanwhile, environmental variables have been shown to potentially change bacterial community composition, likely counteract the impacts of trophic interactions in changing environments [18].

### The effects of environments and bacteria on microeukaryotic plankton community alongside taxonomic resolution

Understanding the microbial community assembly rules and predicting their temporal behavior will be a requisite for developing future protection against water-borne health risks. Ecological network analysis is an effective step to understand and map the correlation between planktons [55]. Our study has established the presence of recurring oscillations of multiple bacterial populations within riverine plankton communities through several annual cycles (Fig. S7). Furthermore, the dynamics of the bacterial community tracked annually repeatable patterns, suggesting community composition can to some extent be evaluated on the basis of environmental factors [56]. In this study, a majority of interactions were between bacteria and protists, with most of these interactions being positive which is the same result as obtained from other planktonic ecosystems [8].

Archaeplastida (e.g. Chlorophyta) provided an opportunity to coexist with heterotrophic microorganisms because of their key role as primary producers [57]. The heterotrophic microorganisms including bacteria were able to decompose organic matter in aquatic environments. Furthermore, their regulation was tied to the capability to sustain microeukaryotic organisms through nutrient interchange and the establishment of intricate communication networks [58]. The close and complex couplings between bacterial and eukaryotic communities across five years that we observed, probably reflects the synchrony or asynchrony in underlying dynamics [59].

The intracellular growth of prokaryotes (including pathogens) in protist cells has been associated with improving their survival in the harsh environment and increase their resistance against antibiotics [60]. The advantage of utilizing protists during in vitro studies is that the research on virulence and pathogenesis of the internalized bacteria can be performed in non-mammalian cells as a model system based on the real situation in nature.

Furthermore, pH played an important role in the community composition of Alveolata, Archaeplastida, Excavata, and Rhizaria taxa (Fig. S8). Human activities may be the main influence on pH values, such as in the Houxi River, which belongs to an urbanizing basin [16]. The downstream environment of the Houxi River is significantly affected by human activities [29], for example, the discharge of domestic sewage and the discharge of acidic or alkaline sewage in industrial processes, is ultimately leading to changes in pH, leading to an imbalance in water acidity and alkalinity and affecting the physiological growth of the four major groups. Nutrients (carbon, nitrogen, and phosphorus) were the main factor significantly affecting Rhizaria and Stramenopiles, showing that the eutrophication level in water quality could significantly alter their community composition.

### Influence of niche-related processes on the microeukaryotic plankton community

Modeling niches at the species level may underestimate the importance of local adaptation processes [5]. The observation of higher explained variance based on sequence similarity rather than database annotation, may indicate that grouping based on database annotation contains more complex ecological significance than the sequence similarity of 97% OTUs, in line with the concept of ecological coherence [61, 62].

Generally, we preferred the finest taxonomic resolution available to evaluate the community data sets over more broad classifications, as the detailed taxonomy is supposed to provide more precise information regarding community composition and niche partitioning patterns [7, 17]. Nevertheless, our findings suggest that there are instances where the utilization of generalized resolution data may be sufficient, or even outperform more detailed classification data, when it comes to discerning the reactions of the planktonic community to environmental factors. In fact, we have been able to find some examples to support the observation that species within the same family constitute niche conservatism, while families within the same phylum constitute significant niche divergence [63, 64].

Adaptations to different ecological niches have shaped and exhibited eukaryotic taxa specialization in evolution, therefore, it is important to acknowledge that these discrepancies should be manifested not solely within the confines of individual species, but also across broader taxonomic categories [61]. Nevertheless, it can often be confirmed that ecological coherence acted on different niches under selective pressures, consistent with bacterial common evolutionary history [65]. Additionally, convergent evolution can also be driven by selective constrains, despite the sharing of different evolutionary histories was generated in bacteria with similar functions [59]. In addition, despite their distinct evolutionary backgrounds, these eukaryotic species exhibit functional similarities. For all these reasons, ecological coherences are expressed as inferring deeper evolutionary divergences and also addressing fundamental evolutionary processes that have shaped such lineages.

However, in species-rich mutualistic networks, the majority of species interact with only a limited number of available partners, thereby restricting direct evolutionary effects [7, 8, 17]. Furthermore, at a higher taxonomic level, middle-taxonomic rank based (91% similarity level) community composition of microeukaryotic community showed highly spatial and temporal patterns in relation to environmental heterogeneity (Fig. 4B). These observations suggest the presence of niche conservatism between 91% and 97% sequence-similarity levels.

Therefore, a graph theory-based analytical model demonstrated that species richness enhances indirect effects in a network by increasing the number of pathways connecting species [66]. Perturbations can alter the adaptive landscape at different scales, leading to changes in network organization, the impact of interactions on evolution, as well as the subsequent evolution of traits [67]. The interconnected nature of multiple interactions may moderate the plankton responses to environmental changes, promoting the persistence of co-evolutionary dynamics. In natural ecosystems, continuous perturbations affect communities, suggesting that networks often deviate from equilibrium and drive gradual co-evolution reshaping selection regimes and species traits locally [5]. However, a major challenge in studying the microeukaryotic plankton community lies in its extensive diversity and our limited knowledge regarding the ecological aspects of each species [61].

Our partitioning method may still have certain limitations, such as using a 1% threshold rather than a 3% threshold, as a higher or finer resolution may be more conducive to a comprehensive understanding of the origin and variation of microeukaryotes. Even if the current threshold is sufficient to highlight that contemporary diversity patterns that come from long-term evolutionary diversity events, are strongly influenced by environmental correlations and the diversity rate of deep phylogenetic lineages [6, 33, 36].

## Conclusion

Our data displayed that systematically investigating microbial plankton community from fine to broad taxonomic resolutions is practical. Our results illustrate: (i) the effect of bacterial diversity on microeukaryotic plankton diversity was higher than the bacterial abundance along the taxonomic rank of eukaryotes, (ii) microeukaryotic plankton community evolutionary constraints were shown to have a greater role by local environmental variables than by bacterial community, (iii) broadening taxonomic resolution strengthened the niche-related signals and reinforced the present notion of ecological consistency in fine eukaryotic branches. We are confident that this methodology has the potential to be utilized with community-environmental data across various ecosystems, including ecological interactions within the microeukaryotic plankton community. By doing so, we were able to gain insights into documented microbial interactions and derive important biological hypotheses from them.

## Supplementary Material

Supplementary_information_ycae026

## Data Availability

All raw sequences have been uploaded in the public NCBI database under the PRJNA381645 for microeukaryotes (18S rRNA gene) and PRJNA383082 for bacteria (16S rRNA gene), respectively. These raw sequences were also stored in the National Omics Data Encyclopedia (NODE) database under the Project IDs: OEP001922 (18S rRNA gene) and OEP004812 (16S rRNA gene).
