# Supplementary material for: Microeukaryotic plankton evolutionary constraints in a subtropical river explained by environment and bacteria along differing taxonomic resolutions
Source: ISME Commun. 2024 Feb 24;4(1):ycae026. doi: 10.1093/ismeco/ycae026 (PMC10980835; doi:10.1093/ismeco/ycae026)
Supplement: Supplementary_information_ycae026 [file supplementary_information_ycae026.docx]

**Journal: ISME Communications**

*Supplementary information of the article:*

**Microeukaryotic plankton evolutionary constraints in a subtropical river explained by environment and bacteria along differing taxonomic resolutions**

Kexin Ren^a,#^, Yuanyuan Mo^a,b,c,#^, Peng Xiao^a,d^, Regin Rønn^a,e^, Zijie Xu^a,f^, Yuanyuan Xue^a^, Huihuang Chen^a,f^, Windell L. Rivera^g^, Christopher Rensing^a,h,^*, Jun Yang^a,^*

^a^ *Aquatic EcoHealth Group, Key Laboratory of Urban Environment and Health, Fujian Key Laboratory of Watershed Ecology, Institute of Urban Environment, Chinese Academy of Sciences, Xiamen 361021, China*

^b^ *Key Laboratory of Urban Environment and Health, Ningbo Urban Environment Observation and Research Station, Institute of Urban Environment, Chinese Academy of Sciences, Xiamen 361021, China*

^c^ *Zhejiang Key Laboratory of Urban Environmental Processes and Pollution Control, CAS Haixi Industrial Technology Innovation Center in Beilun, Ningbo 315830, China*

^d^ *National and Local Joint Engineering Research Center for Ecological Treatment Technology of Urban Water Pollution, College of Life and Environmental Science, Wenzhou University, Wenzhou 325035, China*

^e^ *Department of Biology, University of Copenhagen, Copenhagen DK2100, Denmark*

^f^ *University of Chinese Academy of Sciences, Beijing 100049, China*

^g^ *Pathogen-Host-Environment Interactions Research Laboratory, Institute of Biology, College of Science, University of the Philippines Diliman, Quezon City 1101, Philippines*

^h^ *Institute of Environmental Microbiology, College of Resources and the Environment, Fujian Agriculture & Forestry University, Fuzhou 350002, China*

# These authors contributed equally to this work

*** Corresponding authors:**

E-mail address: jyang@iue.ac.cn (J Yang)

E-mail address: [rensing@iue.ac.cn](mailto:rensing@iue.ac.cn) (C Rensing)

**This supplementary information contains:**

12 Pages

8 Figures

**This file includes:**

**Fig. S1.** Dynamics of environmental variables in the Houxi River from 2012 to 2016.

**Fig. S2.** Microeukaryotic plankton biodiversity calculated following hierarchical classification (from 97% to 70% sequence similarity levels).

**Fig. S3.** Pairwise correlations of abundance-weighted compositions measured at different taxonomic resolutions based on Procrustes analyses.

**Fig. S4.** Spearman correlation between the absolute abundance of microeukaryotic 18S rRNA gene and bacterial 16S rRNA gene from the Houxi River based on qPCR data.

**Fig. S5.** Spearman correlation between microeukaryotic plankton Shannon-Wiener diversity and bacterial diversity or abundance measured at different microeukaryotic plankton groups, taxonomic and phylogenetic resolutions.

**Fig. S6.** Number of edges in interaction network between microeukaryotic plankton taxa (97% similarity level) and bacterial taxa (97% similarity level).

**Fig. S7.** Co-occurrence dynamics of bacterial OTUs and microeukaryotic plankton OTUs along Houxi River stations, showing the relatively abundance of high network degree of OTUs in each taxonomic group, respectively.

**Fig. S8.** Correlation between environmental variables and microeukaryotic plankton in the Houxi River.

**Materials and methods**

*Physical and chemical analyses*

A total of 15 environment variables were included in this study. Seven variables including surface water temperature (WT), dissolved oxygen (DO), pH, chlorophyll *a* (Chl *a*), oxidation-reduction potential (ORP), electrical conductivity (EC), and turbidity were measured *in situ* with a multi-parameter water quality analyzer (Hydrolab DS5, Hach Company, Loveland, CO, USA). Total carbon (TC), total organic carbon (TOC), total nitrogen (TN), ammonium nitrogen (NH_4_-N), nitrate nitrogen (NO_3_-N), nitrite nitrogen (NO_2_-N), total phosphorus (TP) and phosphate phosphorus (PO_4_-P) were determined following our previous procedure (Liu et al., 2013; Isabwe et al., 2022).


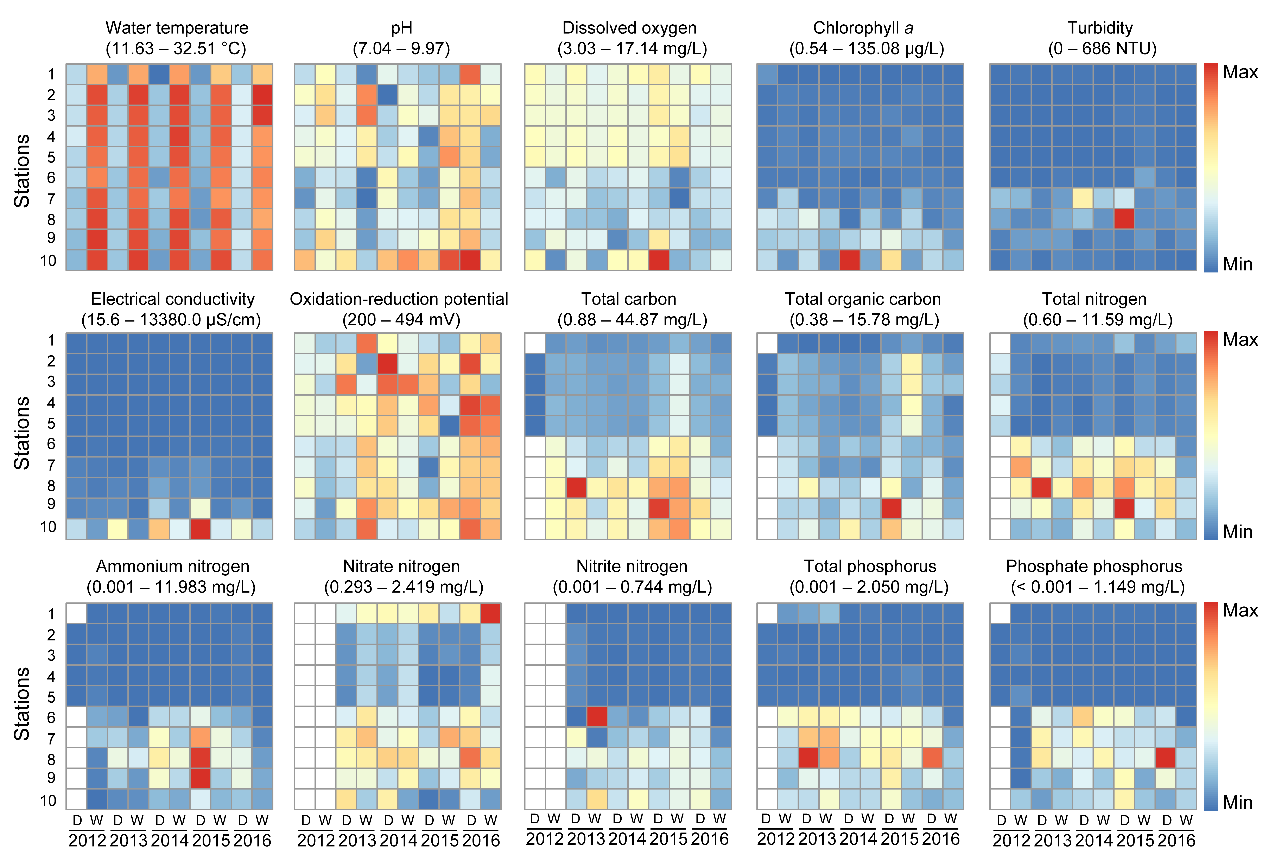


**Fig. S1.** **Dynamics of environmental variables in the Houxi River from 2012 to 2016**. The blue colors represent the lower values, while the red colors represent the higher values. Blank boxes indicate missing values (no data available). D and W represent dry and wet seasons, respectively.


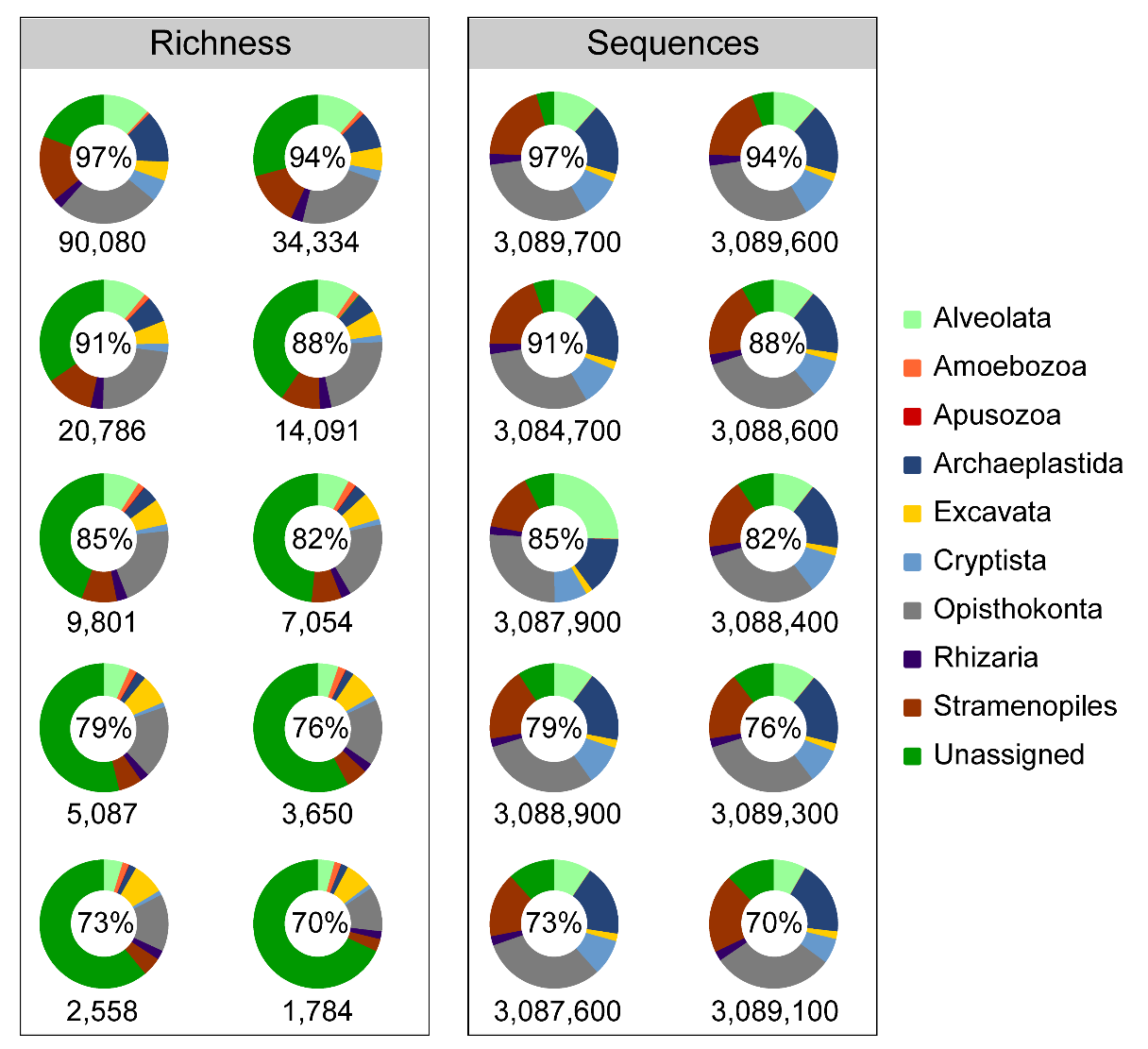


**Fig. S2.** **Microeukaryotic plankton biodiversity calculated following hierarchical classification (from 97% to 70% sequence similarity levels)**. Phylogenetic breakdown of the entire metabarcoding data set at the microeukaryotic supergroup level. All V9 rRNA gene reads and OTUs were classified as belonging to the nine recognized eukaryotic supergroups. “Unassigned” represent OTUs assigned to other taxa or were not assigned with the Usearch SINTAX algorithm with a cut-off of 0.8. The number indicates the number of OTUs and number of sequences in each OTU table, respectively.


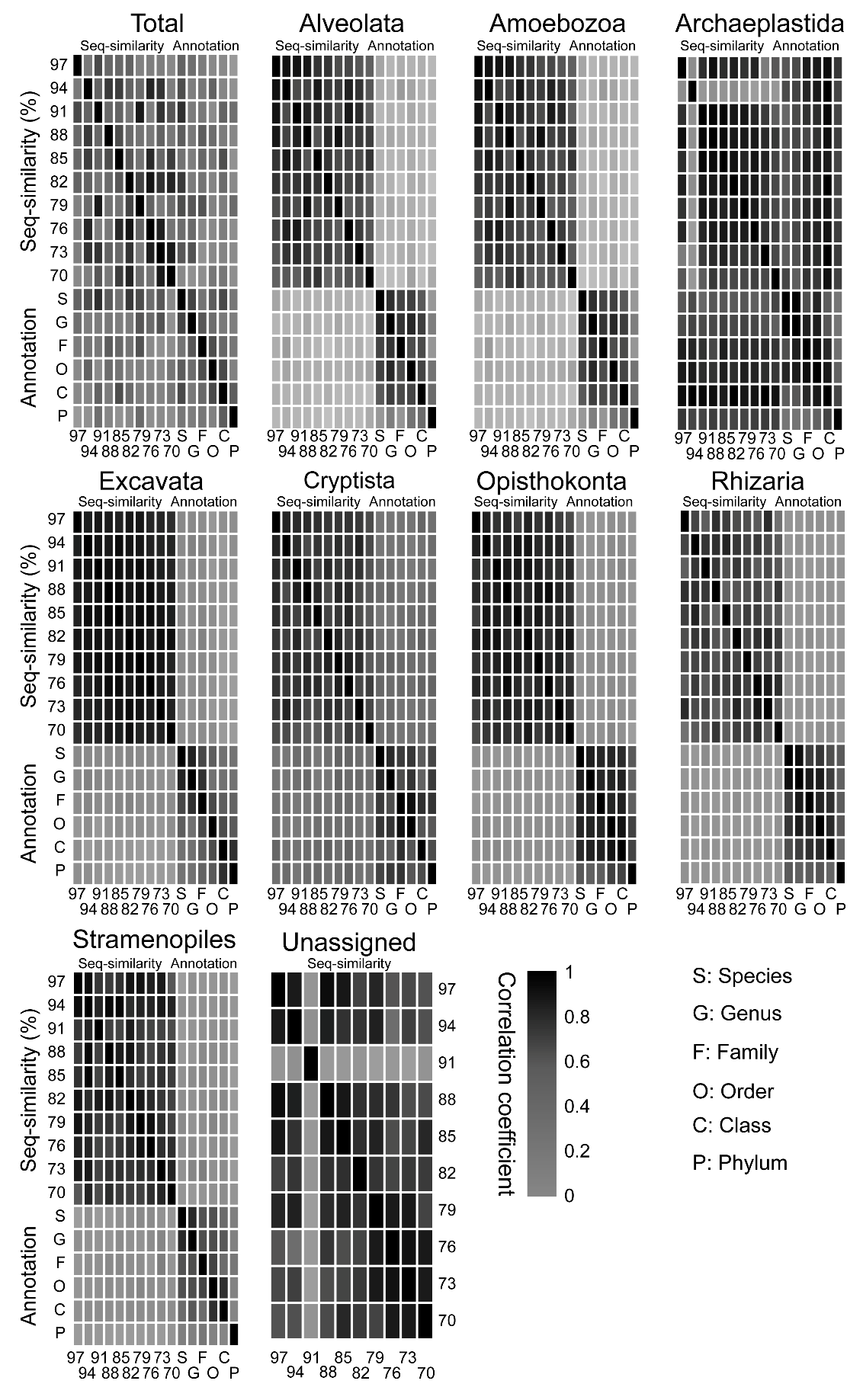


**Fig. S3. Pairwise correlations of abundance-weighted compositions measured at different taxonomic resolutions based on Procrustes analyses.** Measures of taxonomy-based compositions are calculated following two types of hierarchical classification (sequence similarity: from OTU 97% to OTU 70%; and database annotation: from species to phylum). We divided them into nine different components (supergroups) of the microeukaryotic plankton community and calculated the correlation coefficients. 97 to 70 represent the sequence similarity from fine (97%) to broad (70%); and S to P indicate database annotation from species to phylum level.
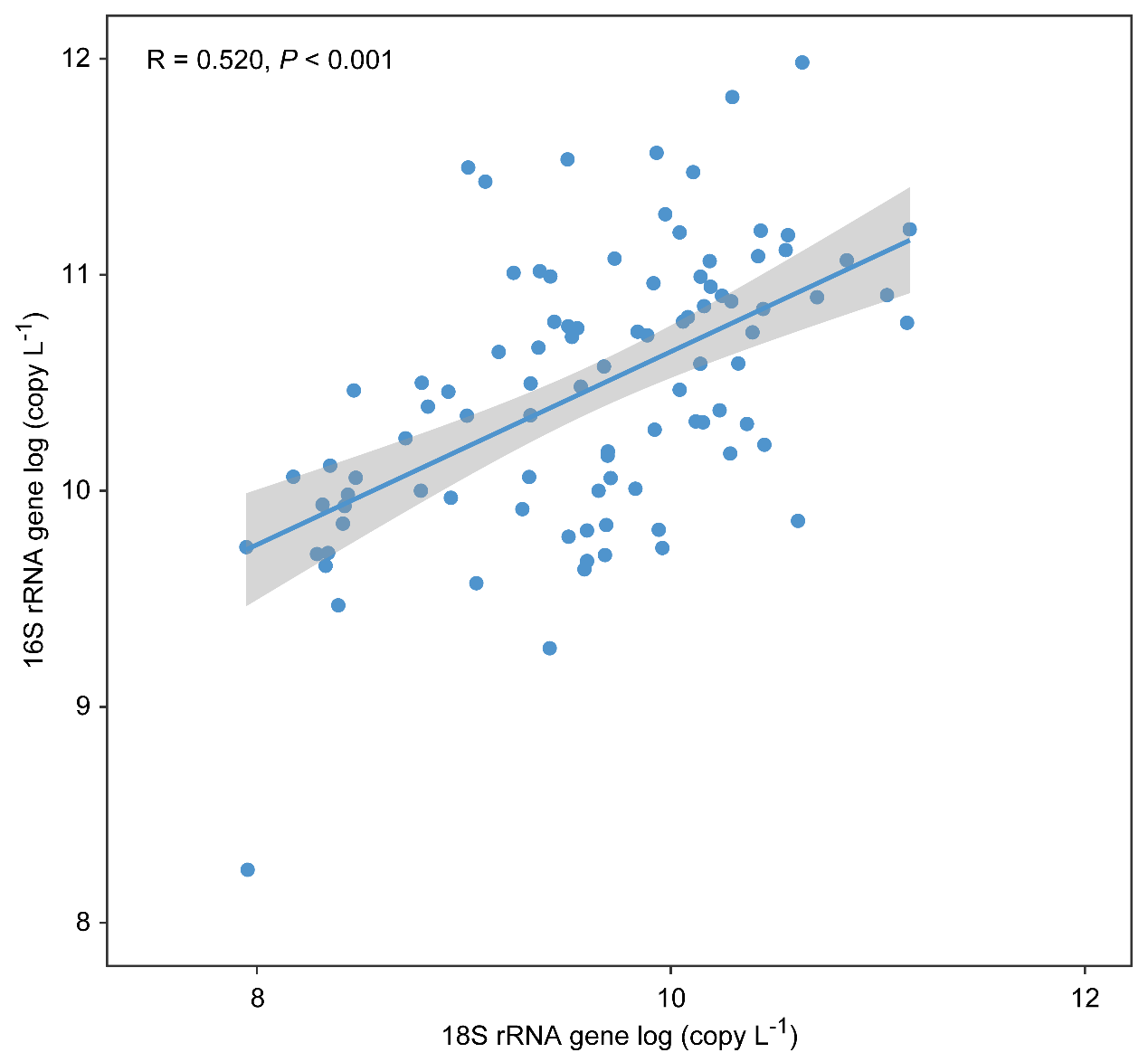


**Fig. S4. Spearman correlation between the absolute abundance of microeukaryotic 18S rRNA gene and bacterial 16S rRNA gene from the Houxi River based on qPCR data.**


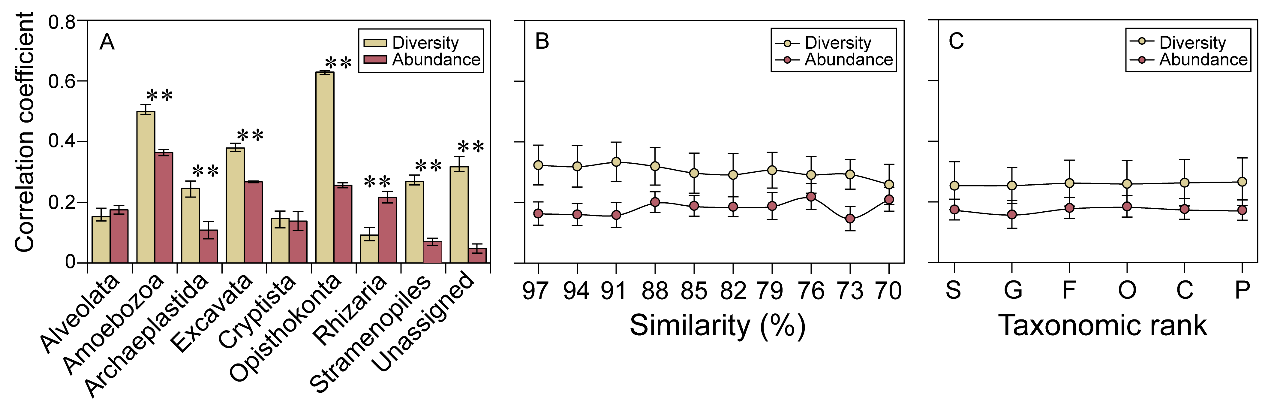


**Fig. S5.** **Spearman correlations between microeukaryotic plankton Shannon-Wiener diversity and bacterial diversity or abundance measured at different microeukaryotic plankton groups, taxonomic and** **phylogenetic resolutions.** (**A**) Bar-plot summarizing the strength of the correlation between the diversity (Shannon-Wiener index) of microeukaryotic plankton supergroups and bacterial diversity (Shannon-Wiener index)/ bacterial abundance (Log 16S gene copies). Asterisks denote significant differences in the strength of the correlation between bacterial OTUs and abundance were determined by two-sample *t*-tests (**P* < 0.05, ***P* < 0.01). (**B**) Measurements of correlation between microeukaryotic plankton Shannon-Wiener index and bacterial Shannon-Wiener diversity or bacterial abundance at different levels of sequence similarity. (**C**) The correlation coefficients between microeukaryotic plankton Shannon-Wiener index and bacterial Shannon-Wiener index or bacterial abundance along the taxonomic rank from species to phylum levels (S: species, G: genus, F: family, O: order, C: class, P: phylum) based on database annotation.


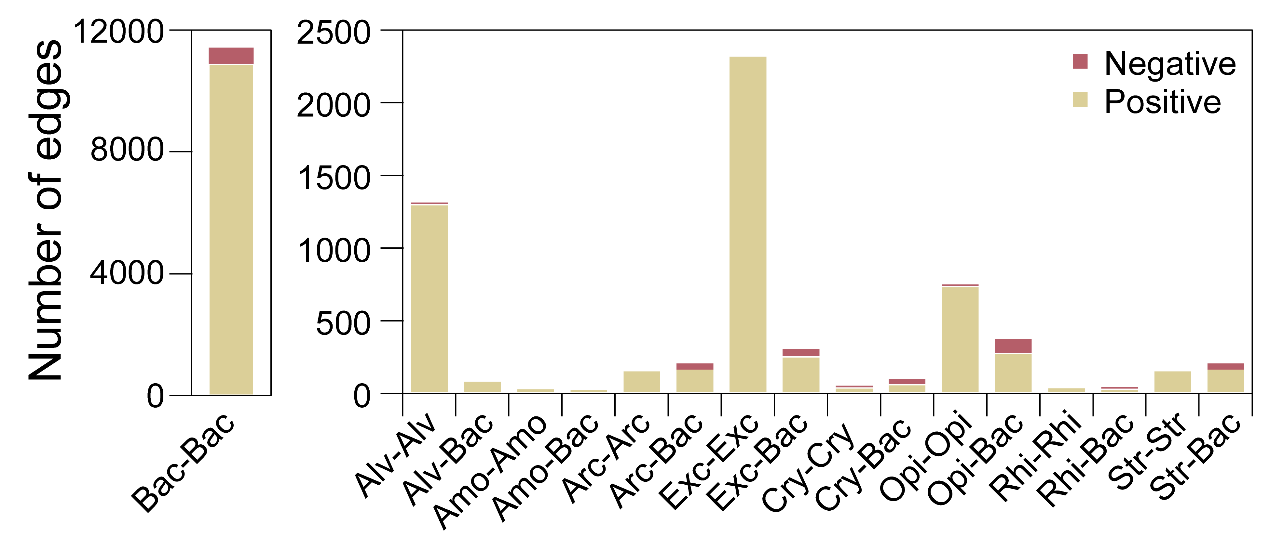


**Fig. S6.** **Number of edges in interaction network between microeukaryotic plankton taxa (97% similarity level) and bacterial taxa (97% similarity level)**. Bac-Bac, Bacteria *vs.* Bacteria; Alv-Alv, Alveolata *vs.* Alveolata; Alv-Bac, Alveolata *vs.* Bacteria; Amo-Amo, Amoebozoa *vs.* Amoebozoa; Amo-Bac, Amoebozoa *vs.* Bacteria; Arc-Arc, Archaeplastida *vs.* Archaeplastida; Arc-Bac, Archaeplastida *vs.* Bacteria; Exc-Exc, Excavata *vs.* Excavata; Exc-Bac, Excavata *vs.* Bacteria; Cry-Cry, Cryptista *vs.* Cryptista; Cry-Bac, Cryptista *vs.* Bacteria; Opi-Opi, Opisthokonta *vs.* Opisthokonta; Opi-Bac, Opisthokonta *vs.* Bacteria; Rhi-Rhi, Rhizaria *vs.* Rhizaria; Rhi-Bac, Rhizaria *vs.* Bacteria; Str-Str, Stramenopiles *vs.* Stramenopiles; Str-Bac, Stramenopiles *vs.* Bacteria. Note that only robust (|r| ≥ 0.6) and statistically significant (*P*-value < 0.01) correlations were incorporated into network analyses.


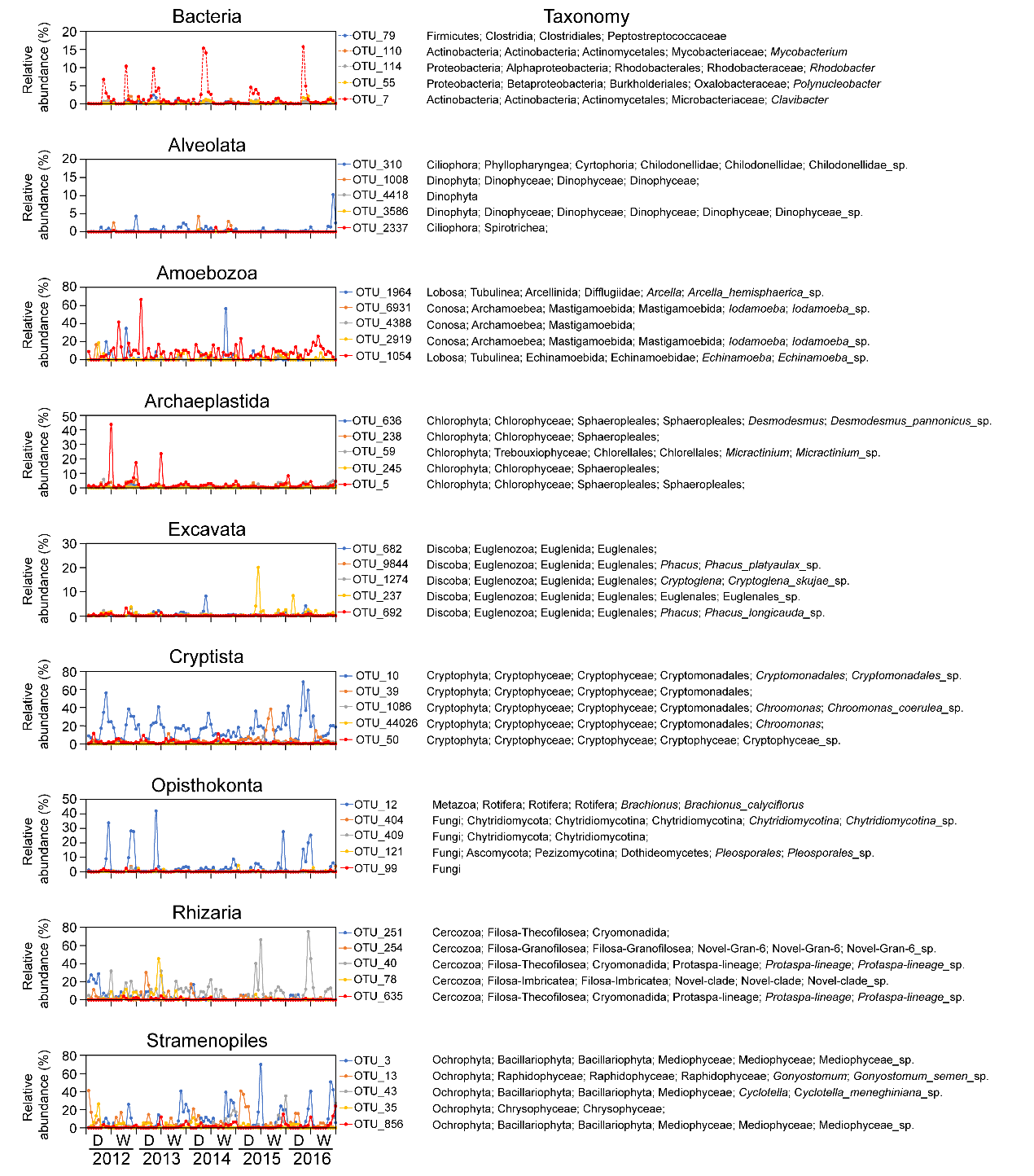


**Fig. S7. Co-occurrence dynamics of bacterial OTUs and microeukaryotic plankton OTUs along Houxi River stations, showing the relatively abundance of high network degree of OTUs in each** **taxonomic group, respectively.** D and W represent dry and wet seasons, respectively.


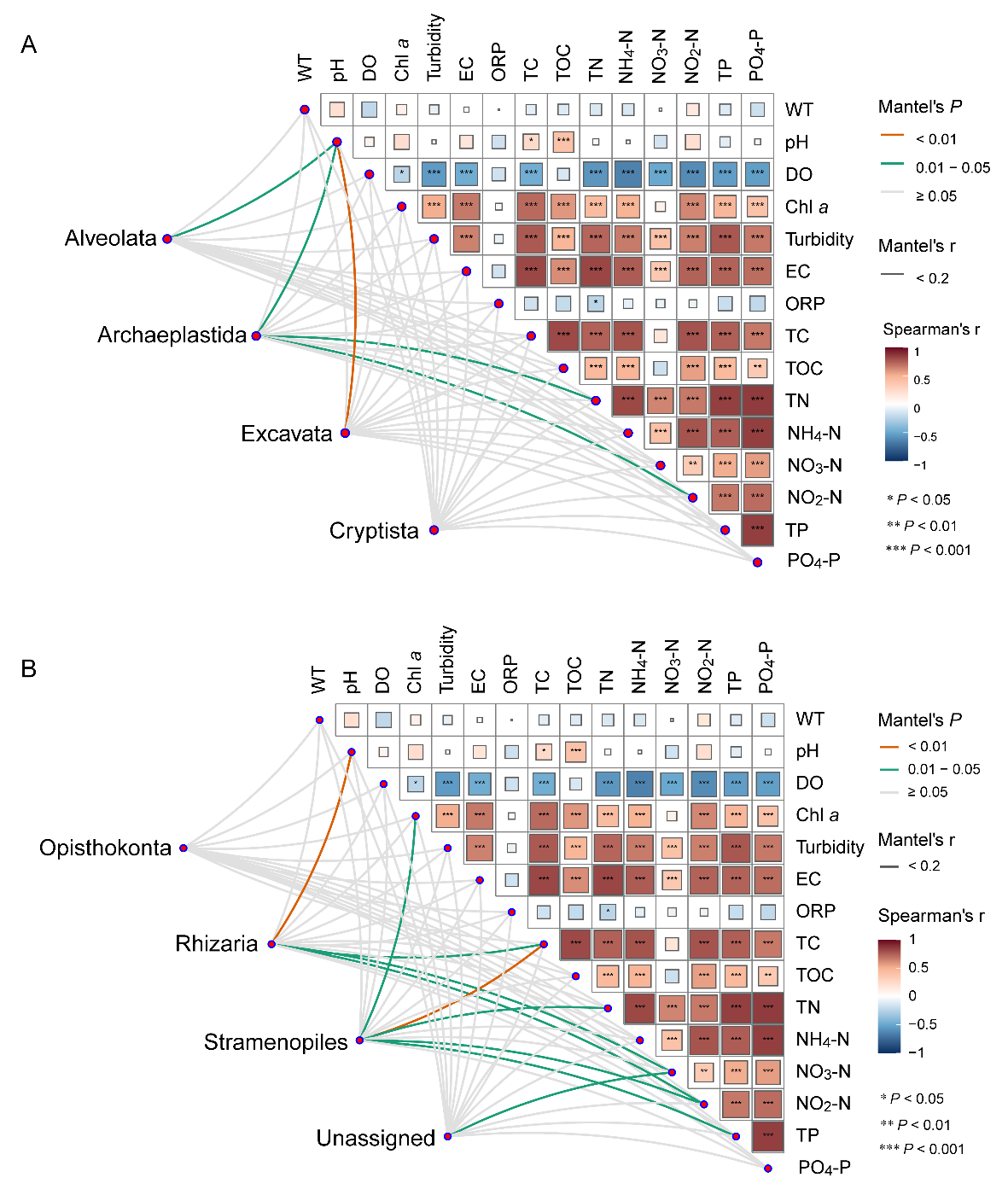


**Fig. S8. Correlation between environmental variables and microeukaryotic plankton in the Houxi River. A**, **B**. The linkage of environmental variables to the eight major microeukaryotic groups from microeukaryotic plankton OTU table based on 97% similarity level by Mantel tests. Pairwise comparisons of environmental variables are shown in the upper-right section, with a color gradient representing Spearman’s correlation coefficients. The line width represents the Mantel’s r statistic for the corresponding correlation, and line color means that significances are tested based on 999 permutations. WT, water temperature; DO, dissolved oxygen; Chl *a*, chlorophyll *a*; EC, electrical conductivity; ORP, oxidation-reduction potential. TC, total carbon; TOC, total organic carbon; TN, total nitrogen; NH_4_-N, ammonium nitrogen; NO_3_-N, nitrate nitrogen; NO_2_-N, nitrite nitrogen; TP, total phosphorus; PO_4_-P, phosphate phosphorus.

**References**

Isabwe, A., Yang, J.R., Wang, Y.M., Wilkinson D.M., Graham E.B., Chen, H.H., Yang, J., 2022. Riverine bacterioplankton and phytoplankton assembly along an environmental gradient induced by urbanization. ***Limnol. Oceanogr.*** 67, 1943–1958.

Liu, L.M., Zhang, D.H., Lv, H., Yu, X.Q., Yang, J., 2013. Plankton communities along a subtropical urban river (Houxi River, southeast China) as revealed by morphological and molecular methods. ***J. Freshwater Ecol.*** 28, 99–112.
